# Autonomous AI-driven point-of-care screening for diabetic retinopathy compared to reading center multi-expert clinical review: results from three prospective controlled pivotal validation studies with AEYE-DS in over 1,200 patients

**DOI:** 10.3389/fdgth.2026.1907804

**Published:** 2026-08-25

**Authors:** Zack Dvey-Aharon, Moshe Livne, Dan Margalit, Amit Wohl, Adi Haupt, Rachelle Aviv, Ahava Stein, Adi Rachelson, Jonathan Nussdorf, Tsontcho Ianchulev

**Affiliations:** 1 AEYE Health Inc., New York, NY, United States; 2 A. Stein – Regulatory Affairs Consulting Ltd., Kfar Saba, Israel; 3 Department of Ophthalmology, Ochsner Health, New Orleans, LA, United States; 4 Queensland Medical Program, University of Queensland, Brisbane, QLD, Australia; 5 New York Eye and Ear of Mount Sinai, Icahn School of Medicine, New York, NY, United States

**Keywords:** artificial intelligence, autonomous screening, clinical trials, diabetic retinopathy, DR screening

## Abstract

**Purpose:**

Diabetic retinopathy (DR) remains the leading cause of blindness among working-age adults and requires regular screening to detect progression among the growing global diabetic population. This study evaluated the performance of AEYE-DS, an autonomous artificial intelligence (AI) system designed for high-throughput, point-of-care analysis of retinal images, in detecting more-than-mild diabetic retinopathy (mtmDR) during routine screening of patients with diabetes who had not previously been diagnosed with DR.

**Principal results:**

AEYE-DS was tested across three prospective clinical studies using two FDA-cleared non-mydriatic retinal cameras: the handheld Aurora and the desktop Topcon NW400. The algorithm autonomously analyzed retinal images and determined mtmDR presence. Diagnostic outcomes were compared to a reference standard based on the Early Treatment for Diabetic Retinopathy Study (ETDRS) severity grading performed by multi-expert review at an independent reading center. Sensitivity and specificity were 93% and 91% in AEYE-1 (95% CI: 83%–97% and 88%–94%), 92% and 94% in AEYE-2 (95% CI: 79%–97% and 90%–96%), and 93% and 89% in AEYE-3 (95% CI: 80%–97% and 85%–92%). Imageability was >99% in all studies. Intra-operator repeatability exceeded 99% for both devices. Between-operator reproducibility was 98% for the desktop camera and 95% for the handheld device, while between-device reproducibility reached 99% and 97%, respectively.

**Conclusions:**

AEYE-DS demonstrated high diagnostic accuracy, imageability, reliability, and reproducibility across different operators and devices in non-mydriatic settings. Findings support autonomous AI system use for scalable, point-of-care DR screening, potentially expanding access, streamlining workflows, and reducing the global burden of diabetic eye disease.

## 1 Introduction

Diabetic retinopathy (DR) screening represents a significant challenge in the care of hundreds of millions of diabetic patients both in the United States and globally (1, 2). DR remains the leading cause of blindness among working-age adults, despite being largely preventable through early detection and intervention (3, 4). Vision loss due to DR is almost entirely avoidable if the disease is identified and managed in its early stages or in many cases even relatively advanced, yet a-symptomatic stages. For this reason, more than 830 million individuals with diabetes in the world (5), and approximately 40 million individuals with diabetes in the United States (6), require annual diabetic eye screening exam. However, studies indicate that even in the developed world more than 50% of patients with diabetes fail to complete the recommended annual eye examination despite this being one of the major health quality HEDIS measures for population health in the US (7), and similar quality measures abroad. While the reasons for this non-adherence are often multi-factorial, a significant challenge pertains to systemic barriers such as access to a timely, affordable and convenient retinopathy screening (8). In the developing world, the social determinants of health are even more pronounced, leading to unnecessarily high burden of diabetic disease (9).

To address this care gap, new, more efficient and scalable programs are needed for high-throughput point-of-care screening. Tele-retina screening services have emerged with point-of-care retinal image acquisition followed by remote expert manual review by clinicians. Studies have shown positive impact of tele-retina centers with increased screening rates (10, 11), but significant challenges remain, both economic and operational. The clinical analysis of the images is laborious and requires highly expert clinicians, often highly trained retina subspecialists. In addition, the images submitted are reviewed days or weeks later and many are deemed of insufficient quality for grading well after the patient encounter. This creates additional logistical and administrative burdens, as follow-up consultations must be coordinated between patients and providers. Furthermore, tele-retina screening often exhibits suboptimal diagnostic performance, particularly in sensitivity, based on the quality of the reviewer.

Artificial intelligence (AI) applications have emerged in recent years in health care, specifically in retinal imaging (12–16).

The application of AI in diabetic retinopathy (DR) screening has the potential to transform the care delivery paradigm by addressing critical limitations of traditional tele-retina services. AI systems can eliminate the reliance on expert readers, which represent the most costly component of tele-retina screening, and provide immediate, on-the-spot image analysis, allowing for the real-time identification and correction of poor-quality images. This instant feedback not only improves screening efficiency but also enables providers to communicate results directly to patients during the same visit, thereby reducing significant downstream administrative burdens.

Early AI applications in DR screening showed tremendous promise but significant challenges prevent their widespread deployment. Diagnostic efficacy below the meaningful threshold of 90%, along with significant usability limitations including low gradability and hardware limitations inhibited practical deployment at a population scale. In 2018, the FDA approved the first autonomous AI-based DR screening solution, IDx-DR, which allowed screenings to be conducted without expert validation (17). While this marked a significant regulatory milestone, the corresponding sensitivity of only 86%–87% and specificity of 89.5% leave significant performance gaps to be addressed. The system also requires multiple high-quality images per eye which can take up to 30 min—not a practical time for the average, busy, primary care clinic. In addition, approximately 25% of patients fail non-mydriatic acquisition and require pharmacologic dilation—which can only be done by eye care providers such as ophthalmologists and optometrists, also due to high liability risks (e.g., glaucoma). This nullifies one of the main health economic value drives of the system as a screening tool at the point of primary care interaction with the patient. The system is also non-portable and limited to desktop camera equipment, limiting the real-world use to a minority of point of care locations. Similarly, in 2020, there was a second FDA clearance of the EyeArt (Eyenuk, Inc.) with very similar diagnostic profile and limitations. Several years following the approval of those two autonomous systems for diabetic retinopathy screening, the footprint of these technologies in the field is minimal, impacting less than 0.1% of all screenings in the US despite available reimbursement and HEDIS quality measure incentives (18).

AEYE-DS represents a next-generation AI using sophisticated, novel deep learning methodology to increase diagnostic efficacy, precision and imageability while remaining camera agnostic so that it can meet the requirements for practical point-of-care deployment and high-throughput processing. AEYE-DS is designed to be camera agnostic, compatible with both portable and tabletop imaging devices, relies on only one image per eye and rarely requires dilation. This versatility significantly expands the reach of DR screening beyond traditional clinical settings.

Three prospective clinical validation trials in over 1,200 diabetic patients were used for the two FDA approvals of the technology. Herein we report the results of those studies.

## 2 Methods

Three H2H clinical studies of AI-based autonomous DR screening with the AEYE-DS system vs. gold-standard reading center diagnostic performance were designed for the clinical validation of the camera agnostic autonomous system.

### 2.1 Study population

The study population included subjects with a confirmed diagnosis of diabetes who have not been previously diagnosed with diabetic retinopathy. Diabetes was diagnosed according to criteria established by the World Health Organization (WHO) or the American Diabetes Association (ADA). These diagnostic criteria included:

- Hemoglobin A1c (HbA1c) ≥6.5%, based onrepeated assessments
- Fasting Plasma Glucose (FPG) ≥126 mg/dL (7.0 mmol/L), based on repeated assessments
- Oral Glucose Tolerance Test (OGTT) with 2-hour plasma glucose (2-h PG) ≥200 mg/dL (11.1 mmol/L) using a 75 g oral anhydrous glucose dose dissolved in water
- Random plasma glucose (RPG) ≥200 mg/dL (11.1 mmol/L) in the presence of symptoms of hyperglycemia or hyperglycemic crisis

Participants underwent routine retinal screening for diabetic retinopathy (DR) in various healthcare settings, including hospitals, primary care clinics, and medical research centers. Subjects were of both genders, diverse ethnic backgrounds, and were aged ≥22 years. Recruitment efforts ensured a representative sample of the intended use population for the device—for each study, a sample size calculation was based both on the statistical analysis for the success criteria, as well as the expected prevalence of DR among the screened individuals.

Exclusion criteria are detailed in Table 1.

**Table 1:** Exclusion criteria.

- Uncorrectable vision loss (e.g., with the use of eyeglasses), blurred vision, or floaters; - Diagnosed with macular edema, severe non-proliferative retinopathy, proliferative retinopathy, radiation retinopathy, or retinal vein occlusion; - Previously diagnosed with Diabetic Retinopathy; - History of laser treatment of the retina or injections into either eye, or any history of retinal surgery; - Currently participating in another investigational eye study and actively receiving investigational product for DR or DME; - A condition that, in the opinion of the investigator, would preclude participation in the study; - Contraindicated for imaging by fundus imaging systems used in the study because of hypersensitivity to light, recently underwent photodynamic therapy (PDT), taking medication that causes photosensitivity or has a history of angle-closure glaucoma or narrow anterior chamber angles; - Pregnancy

### 2.2 AEYE-DS artificial intelligence algorithm

**Figure 1:**
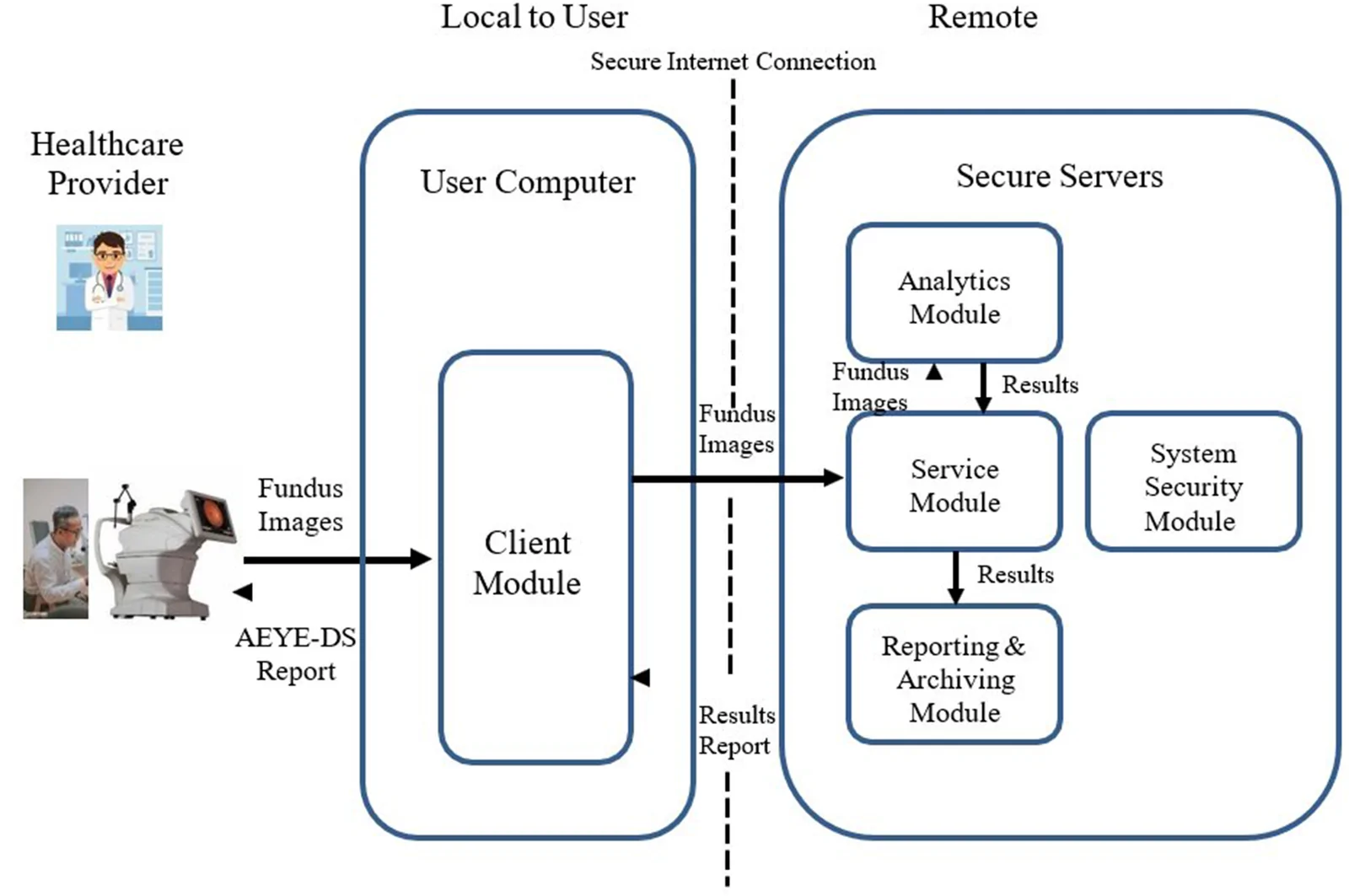
AEYE-DS device configuration.

AEYE-DS is a retinal diagnostic software-as-a-service device that incorporates an algorithm to evaluate retinal images for diagnostic screening to identify retinal diseases or conditions. Specifically, the AEYE-DS is designed to perform diagnostic screening for the condition of more-than-mild diabetic retinopathy (mtmDR). The AEYE-DS is comprised of 5 software components: (1) Client; (2) Service; (3) Analytics; (4) Reporting and Archiving; and (5) System Security (Figure 1).

The device configuration of these modules is presented in Figure 1 below, indicating which components are local to the user and which are remotely located. The fundus camera is attached to a computer, where the Client module/software is installed. The Client module/software guides the user to acquire the images and enables the user to interact with the server-based analysis software over a secure internet connection. Using the Client module/software, users identify the fundus images per eye to be dispatched to the Service module/software. The Service module/software is installed on a server hosted at a secure datacenter, receives the fundus images and transfers them to the Analytics module/software. The Analytics module/software, which runs alongside the Service module/software, processes the fundus images and returns information on the image quality and the presence or absence of mtmDR to the Service module/software. The Service module/software then returns the results to the Client module/software.

### 2.3 Patient data collection

The FDA validation dataset comprises three prospective head-to-head validation clinical studies of the AEYE-DS technology in a total of 1,210 patients. The study protocols adhered to the tenets of the Declaration of Helsinki and were approved by the Sterling central institutional review board, as well as registered on clinicaltrials.gov (NCT06241664 and NCT05857943).

General patient demographics, medical history, concomitant medications, and imaging system details were recorded for each study participant on a standardized Case Report Form (CRF). Ocular imaging was performed by novice operators with no prior experience in retinal imaging. Each operator captured a single macula-centered fundus photograph from each eye using the Topcon Model NW400 desktop fundus camera and/or the Optomed Aurora handheld fundus camera.

### 2.4 Image acquisition and submission

Captured fundus images were submitted to the AEYE-DS client software for analysis. If the AEYE-DS software determined that one or more images were of insufficient quality (IQ), the novice operator retook the required images and resubmitted them. A maximum of three attempts was permitted during the initial imaging session. If image quality remained insufficient after three attempts, tropicamide 1.0% eyedrops were administered for pupil dilation, and the operator was allowed an additional three attempts. If all six attempts failed to produce sufficient image quality, the participant was categorized as having insufficient quality images.

### 2.5 Diagnostic output

For cases where images met quality standards, the AEYE-DS system produced a diagnostic output of either “more-than-mild diabetic retinopathy (mtmDR) detected” or “mtmDR not detected,” accompanied by a PDF diagnostic report. For participants with insufficient image quality after six attempts, no diagnostic result was generated. Following the AEYE-DS assessment, all participants underwent professional retinal imaging for validation.

### 2.6 Professional imaging and reference standard

Professional retinal imaging for each eye included four dilated stereo widefield fundus photography, lens photography to assess media opacity, and macular optical coherence tomography (OCT) imaging. Tropicamide 1.0% was used for pupil dilation unless the participant’s pupils were already dilated during the novice imaging phase.

All professional images were submitted to an independent, certified reading center where retinopathy severity and diabetic macular edema (DME) were graded according to the Early Treatment Diabetic Retinopathy Study (ETDRS) severity scale. Diagnoses were determined using a multimodal approach, incorporating both fundus and OCT images as well as fundus-only based approach.

### 2.7 Reader masking and evaluation

Three certified readers, masked to the AEYE-DS outputs, independently reviewed the images. Masking protocols ensured that fundus image evaluations were blinded to OCT findings and vice versa. Diagnostic consensus was achieved using a majority voting paradigm. The Reading Center classified each participant’s retinopathy as either mtmDR + or mtmDR− based on the more severely affected eye. The definition of mtmDR + was presence of either retinopathy with ETDRS score ≥35, or clinically significant DME (CSDME).

### 2.8 Outcome comparison and statistical analysis

The AEYE-DS diagnostic results were compared with the reference standard diagnosis determined by the Reading Center. Sensitivity and specificity were calculated at the participant level for both multimodal (fundus + OCT) and fundus-only diagnoses. Statistical analyses were conducted to evaluate the concordance between the AEYE-DS output and the reference standard.

### 2.9 Human factors assessment

The usability of the AEYE-DS system was evaluated through human factors validation studies conducted for each camera used in conjunction with the system. These studies assessed comprehension of the User Manual and overall usability of the device by novice operators. Human factors studies were performed at one of the study sites under separate, predefined protocols specifically designed to test usability in real-world scenarios.

The validation protocols focused on the AEYE-DS device, including camera operation, adherence to the imaging protocol, accurate interpretation of device outputs, and effective use of the standardized training materials. These protocols were aligned with the FDA Guidance Document, which recommends a minimum sample size of 15 users to effectively identify user interface and design issues (19).

Approximately 15 novice operators participated in each human factors study. All participants completed a one-time standardized training program that covered image acquisition techniques, strategies for improving image quality following an “insufficient quality” output from the AEYE-DS system, and the process for submitting images for analysis. After completing the training, participants were given a 1-hour break before performing the use-case tasks under direct observation. These tasks were designed to simulate real-world operation of the system to evaluate its usability under practical conditions.

### 2.10 Precision sub-studies

The repeatability and reproducibility of the AEYE-DS diagnostic outputs were evaluated through precision studies conducted at one of the study sites. These studies followed predefined protocols designed to assess agreement across multiple measurements obtained from various users under controlled conditions.

Repeatability was defined as the precision of measurements obtained under identical operating conditions over a short period of time, while reproducibility assessed the precision of measurements obtained under varying conditions, including different operators and imaging devices.

Each precision study included approximately 10 participants diagnosed as “mtmDR−” and 10 participants diagnosed as “mtmDR+” based on grading by the Expert Reading Center. Novice operators imaged participants using the AEYE-DS imaging protocol, capturing 12 consecutive diagnostic outputs per participant. Each participant was imaged by three different novice operators using two different cameras of the same type (either the Topcon NW400 desktop camera or the Optomed Aurora handheld camera).

This resulted in 12 image sets per participant and 264 image sets in total across the study. These diagnostic outputs were analyzed to evaluate precision metrics under both repeatability and reproducibility conditions, providing a comprehensive assessment of the AEYE-DS system's consistency across varying operational scenarios.

## 3 Results

### 3.1 Patient accountability

The first pivotal study (AEYE-1) was conducted across eight clinical sites, including seven in the United States and one in Israel, with active patient enrollment occurring between October 2020 and November 2021. A total of 531 participants were screened.

A second clinical study (AEYE-2) comprising a subset of 317 AEYE-1 study subjects that underwent additional screening utilizing the Optomed Aurora handheld fundoscopy camera.

The handheld-focused study (AEYE-3) was conducted at five clinical sites within the United States, with active enrollment between March 2023 and September 2023. In this study, 365 participants were screened.

**Figure 2:**
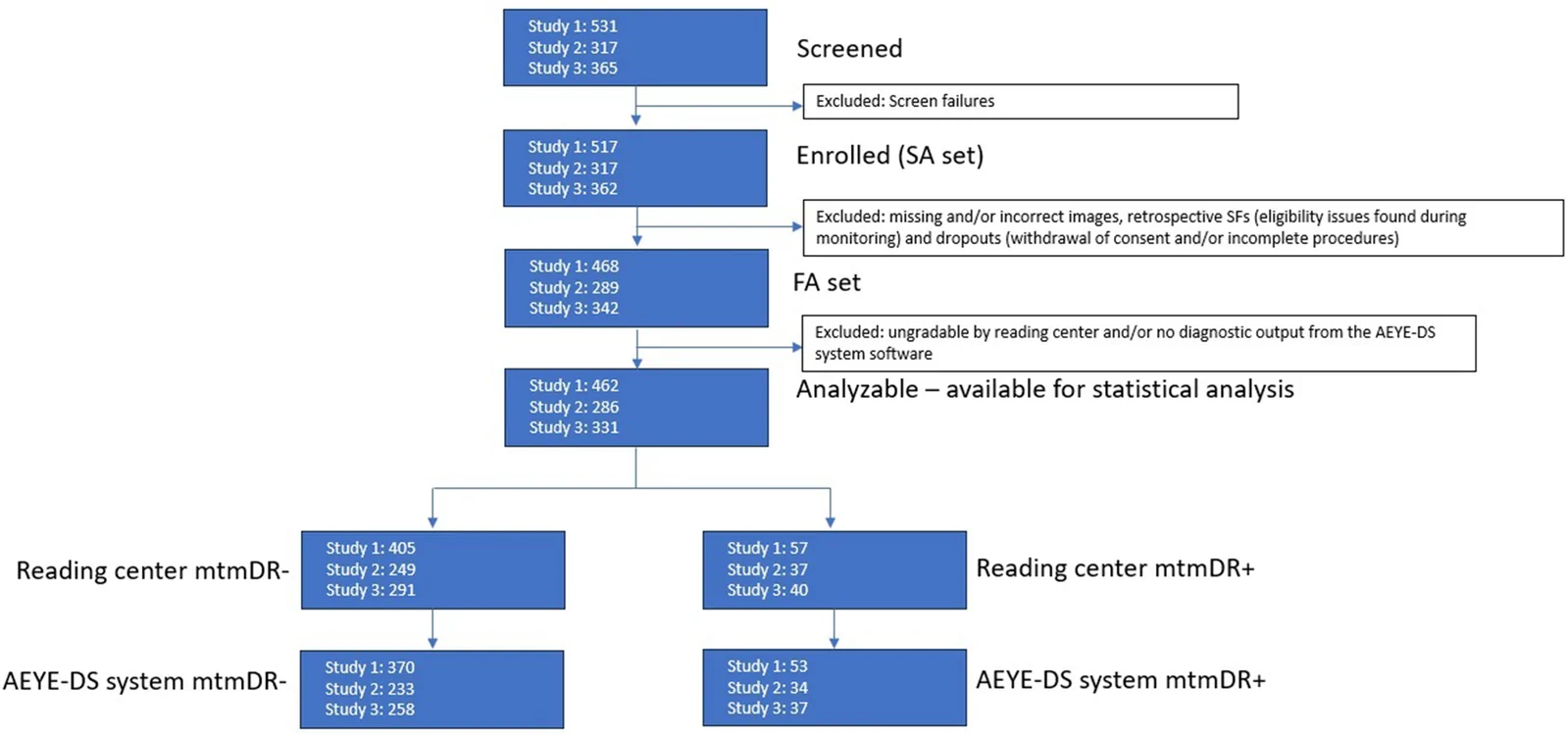
Consort Diagram for the three studies.

A CONSORT diagram illustrating the final disposition of all study subjects across the screened, enrolled, safety analysis (SA), and full analysis (FA) populations is presented in Figure 2. The SA population was used as the primary dataset for the analysis of patient demographics and baseline characteristics, while the FA population served as the primary dataset for evaluating both the primary and secondary efficacy endpoints, as a small number of patients were not able to be screened.

### 3.2 Demographic and baseline characteristics

The demographic and baseline clinical characteristics for the three studies (AEYE-1, AEYE-2, and AEYE-3) are summarized in Table 2.

**Table 2:** Baseline demographic and clinical characteristics.

Category	Sub-group	Study AEYE-1 (*N* = 517)	Study AEYE-2 (*N* = 317)	Study AEYE-3 (*N* = 362)
Age	Mean (years ± SD)	55 ± 11.8	55 ± 11.5	58 ± 12.2
Sex	Male (%)	47	51	45
	Female (%)	53	49	55
Race	African-American (%)	29	26	19
	Caucasian (%)	39	47	46
Ethnicity	Hispanic or Latino (%)	29	22	30
Duration of diabetes since diagnosis	Average (years ± SD)	10 ± 7.9	10 ± 8	10 ± 8
HbA1c	Average (%± SD)	8.3 ± 2.15	8.2 ± 2.1	8.3 ± 2.2
Diabetes	Type I (%)	5	6	3
	Type II (%)	95	94	97

In the pivotal study (AEYE-1), the mean age of participants was 55 years (SD = 11.8). The study population comprised 47% male and 53% female subjects, with racial/ethnic distribution as follows: 29% African-American, 39% White, 29% Hispanic or Latino, and 3% of other racial/ethnic origins. Approximately 95% of participants were diagnosed with type 2 diabetes, while 5% had type 1 diabetes. The duration of diabetes since diagnosis ranged from 1 day to 44 years, with a mean duration of 10 years (SD = 7.9). The mean hemoglobin A1c (HbA1c) level was 8.26% (SD = 2.15).

For the study AEYE-2, the mean age of participants was also 55 years (SD = 11.8). The cohort consisted of 51% male and 49% female participants, with a racial/ethnic distribution of 26% African-American, 47% White, 22% Hispanic or Latino, and 5% of other racial/ethnic origins. Of the participants, 94% were diagnosed with type 2 diabetes, while 6% were diagnosed with type 1 diabetes. The mean duration of diabetes was 10 years (SD = 8), and the mean HbA1c level was 8.2% (SD = 2.1).

In the second handheld study (AEYE-3), the mean age of participants was 58 years (SD = 12.8). The study population included 45% male and 55% female subjects, with racial/ethnic distribution as follows: 19% African-American, 46% White, 30% Hispanic or Latino, and 5% of other racial/ethnic origins. Approximately 97% of participants were diagnosed with type 2 diabetes, and 3% with type 1 diabetes. The mean duration of diabetes was 10 years (SD = 8), with a mean HbA1c level of 8.3% (SD = 2.2).

### 3.3 Efficacy analysis summary

The AEYE-DS system demonstrated high efficacy in detecting more-than-mild diabetic retinopathy (mtmDR) across three clinical studies, achieving sensitivity and specificity rates exceeding predefined performance goals.

In AEYE-1, sensitivity was 92.98% [CI: 83.30%; 97.24%] and specificity was 91.36% [CI: 88.22%; 93.72%], surpassing the study performance goals of 82% and 87%, respectively. Positive Predictive Value (PPV) was 60.23%, influenced by the natural prevalence of mtmDR+ (12.3%), and Negative Predictive Value (NPV) was 98.93%. Imageability, reflecting the proportion of images successfully analyzed, was 99.14% [CI: 97.81%; 99.67%]. Pharmacologic dilation was avoided in 88.25% of participants, and no significant demographic or clinical variables (e.g., sex, race/ethnicity, age, HbA1c, or diabetes duration) influenced sensitivity or specificity.

AEYE-2, conducted with the handheld Optomed Aurora camera, reported sensitivity of 92% [CI: 79%; 97%] and specificity of 94% [CI: 90%; 96%]. PPV and NPV were 68.00% and 98.73%, respectively, with an imageability rate of 99.31% [CI: 97.50%; 99.81%]. Similar to AEYE-1, no significant effects of clinical or demographic factors on diagnostic performance were observed, and 90% of participants did not require pharmacologic dilation.

AEYE-3, also using the Optomed Aurora handheld camera, confirmed the findings from Study AEYE-2. Sensitivity was 93% [CI: 80%; 97%] and specificity was 89% [CI: 85%; 92%]. PPV was 53%, and NPV was 99%. Imageability was 99% [CI: 96.97%; 99.53%], and pharmacologic dilation was avoided in 90% of participants. Age was the only variable showing a subgroup effect, with younger participants (<55 years) achieving slightly better results.

Across all studies, the AEYE-DS system successfully identified 100% of participants with ETDRS level 43 or higher, ensuring safety of the technology for patients at high risk of vision loss, and demonstrating robust diagnostic accuracy and usability across diverse imaging setups and patient populations.

Table 3 shows the efficacy results of AEYE-DS for mtmDR detection in the camera systems—Topcon NW400 desktop camera and Optomed Aurora handheld camera.

**Table 3:** AEYE-DS diagnostic accuracy in handheld and tabletop cameras.

	AEYE-1	AEYE-2	AEYE-3
	Topcon NW400 Tabletop Camera	Optomed Aurora Handheld Camera	Optomed Aurora Handheld Camera
Sensitivity	93% [83%; 97%]	92% [79%; 97%]	93% [80%; 97%]
Specificity	91% [88%; 94%]	94% [90%; 96%]	89% [85%; 92%]
PPV	60% [50%; 70%]	68% [54%; 79%]	53% [41%; 64%]
NPV	99% [97%; 99%]	99% [96%; 100%]	99% [97%; 100%]
Imageability	99% [98%; 100%]	99% [98%; 100%]	99% [97%; 100%]

### 3.4 Human factors (usability) analysis

Fifteen novice operators using the Topcon NW400 and seventeen using the Optomed Aurora cameras successfully completed all imaging tasks with 100% task completion rates, including critical tasks, following training. All users captured medical-grade retinal images and completed the imaging protocol without errors that could cause harm. Usability feedback indicated that the AEYE-DS system was user-friendly, with difficulties mainly attributed to limited computer skills or inexperience. The user manual was deemed clear and effective by all participants.

### 3.5 Precision analysis

The precision studies included 22 participants with the Topcon NW400 (13 mtmDR−, 9 mtmDR+) and 21 participants with the Optomed Aurora (11 mtmDR−, 10 mtmDR+), generating 264 and 250 image sets/diagnoses, respectively. Intra-operator repeatability was 99% in both studies. Between-operator reproducibility was 98% for the Topcon NW400% and 95% for the Optomed Aurora. Between-device reproducibility was 99% and 97% for the Topcon NW400 and Optomed Aurora, respectively. The high reproducibility across operators and devices highlights the consistency and reliability of the AEYE-DS system. Full agreement results are summarized in Table 4.

**Table 4:** Precision study results.

	Precision results	
	Topcon NW400	Optomed Aurora
Intra-operator repeatability overall agreement	98.48%	99.20%
Between-operator reproducibility overall agreement	97.73%	95.12%
Between-device reproducibility overall agreement	98.48%	96.77%

## 4 Discussion

The results of these studies demonstrated that the AEYE-DS system can perform at a high level of diagnostic efficacy compared to the gold standard multi-expert reading center in a fully autonomous, rapid screening process at the point-of-care for primary care practices. The study demonstrates diagnostic efficacy which exceeded the pre-specified primary endpoint goals with a sensitivity of 92%–93% (>82%), a specificity of 89%–94% (>87%) and an imageability rate of >99%. These results were supportive for the FDA clearance of the AEYE-DS device (20, 21).

AEYE-DS was cleared for use as a fully autonomous real-time diagnostic AI. Given that there is no need for a clinician to interpret the images, the system demonstrates a real-time diagnostic solution to barriers preventing effective, accessible screening for DR. As the leading cause of blindness in the US working age population, as well as many other countries, DR constitutes a large and growing threat to the sight of millions of patients with diabetes.

Comparative review of similar pivotal data of earlier first-generation FDA-cleared AI technologies indicates that DR screening with AEYE-DS on the Topcon NW400 desktop fundoscopy camera (Study AEYE-1) has a higher diagnostic sensitivity at 93% vs. what is reported for the IDX-DR at 85.9%–87% when used in conjunction with the exact same tabletop camera. This is also the case for the specificity metric (91.4% vs. 89.5%). The AEYE-DS imageability of over 99% is a significant improvement over prior systems such as IDX-DŔs of 96%. This is sustained with the handheld device as well where AEYE-DS has an average sensitivity of 93% and specificity of 91.5%. Prior attempts of other technologies with handheld AI-enabled cameras have been challenged by dramatically lower performance rates as reported with RetinaVue700 (Baxter Healthcare, Inc.) featuring modest efficacy of 81% sensitivity (76.6% sensitivity in patients with vision threatening DR) and 82% specificity (22).

AEYE-DS also presents a number of usability-related innovations, when compared to the older-generation devices (e.g., IDx-DR or EyeArt), that make AI screening practical.

The AEYE-DS screens the patient within 1–2 min, using only one image per eye and rarely requires dilation. Point of care environments simply cannot afford lengthy procedures. The results of the AEYE-DS studies have demonstrated that one image per eye is sufficient, and with minimal dilation requirements, even a mediocre image quality can be used for analysis, as shown by the high imageability (>99%).

While tabletop cameras are used regularly in eyecare clinics, they occasionally present real challenges for screening at point of care practices, due to high cost, lack of space and portability. The solution may be found in small, handheld cameras which may be used with AI diagnostic screening devices. The studies confirmed the efficacy of the AEYE-DS with both portable and tabletop cameras.

Limitations of the studies include the relatively small sample size in Study AEYE-2, although the subsequent handheld Study AEYE-3 supplemented the required data to ensure the robustness of the study results with the handheld camera.

The combination of the robust efficacy of the AEYE-DS with a quick and simple procedure with different types of cameras—both portable and stationary—renders AEYE-DS as the first real practical solution for diabetic patient screening at point of care environments. Future studies may evaluate screening adherence improvement in the diabetic patient population, exam time and economics.

## Data Availability

The datasets presented in this article are not readily available because they include proprietary data. Requests to access the datasets should be directed to info@aeyehealth.com.
